# Artificial Evolution by Viability Rather than Competition

**DOI:** 10.1371/journal.pone.0086831

**Published:** 2014-01-29

**Authors:** Andrea Maesani, Pradeep Ruben Fernando, Dario Floreano

**Affiliations:** Laboratory of Intelligent Systems (LIS), Ecole Polytechnique Fédérale de Lausanne (EPFL), Lausanne, Switzerland; University of Vermont, United States of America

## Abstract

Evolutionary algorithms are widespread heuristic methods inspired by natural evolution to solve difficult problems for which analytical approaches are not suitable. In many domains experimenters are not only interested in discovering optimal solutions, but also in finding the largest number of different solutions satisfying minimal requirements. However, the formulation of an effective performance measure describing these requirements, also known as fitness function, represents a major challenge. The difficulty of combining and weighting multiple problem objectives and constraints of possibly varying nature and scale into a single fitness function often leads to unsatisfactory solutions. Furthermore, selective reproduction of the fittest solutions, which is inspired by competition-based selection in nature, leads to loss of diversity within the evolving population and premature convergence of the algorithm, hindering the discovery of many different solutions.

Here we present an alternative abstraction of artificial evolution, which does not require the formulation of a composite fitness function. Inspired from viability theory in dynamical systems, natural evolution and ethology, the proposed method puts emphasis on the elimination of individuals that do not meet a set of changing criteria, which are defined on the problem objectives and constraints.

Experimental results show that the proposed method maintains higher diversity in the evolving population and generates more unique solutions when compared to classical competition-based evolutionary algorithms. Our findings suggest that incorporating viability principles into evolutionary algorithms can significantly improve the applicability and effectiveness of evolutionary methods to numerous complex problems of science and engineering, ranging from protein structure prediction to aircraft wing design.

## Introduction

Evolutionary algorithms are heuristic optimization methods inspired by natural evolution [1]–[4]. They operate by selecting, reproducing, and mutating the genotypes of individuals with higher performance in a population where each individual is a candidate solution to the problem. A fitness function is used to score individuals according to how well they perform on the problem objectives, and a selection operator allocates higher number of copies with random mutations to individuals with higher fitness. This process of fitness-based selection models natural competition between organisms of a population for contributing offspring to the next generation. The generational cycle of fitness assessment, selective reproduction of individuals with higher fitness, and random mutations is repeated until a satisfactory solution to the problem is found. The simplicity, effectiveness, and wide applicability of evolutionary algorithms have contributed to their adoption in a very large number of problem domains, from computer science to engineering, all the way to pharmacology [5], [6]. Moreover, evolutionary algorithms are widely used to investigate biological questions by conducting in-silico experiments [7]–[10].

Evolutionary algorithms are typically employed to discover optimal solutions with respect to one or multiple objectives (single- or multi-objective optimization), that may be subject to constraints (constrained optimization). However, for many real-world problems the objectives or constraints cannot be easily formalized, are computationally too expensive to evaluate and are therefore not used in the search, or can only be approximated [11] (e.g., in evolutionary synthesis of molecular structures [12]). Thus, as the information to discover the real optimal solutions can be missing from the computational problem formulation, one may be interested in finding the largest number of different solutions that satisfy acceptable problem requirements, heuristically choosing the preferred solution(s) once the search process is completed.

In the general case where these requirements are expressed on all the objectives, the problem being solved can be formulated as constraint satisfaction problem. As formulated in [13], a constraint satisfaction problem is a pair 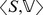, where 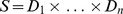 is the search space, 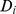 is the allowed set of values for the decision variable 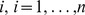, and 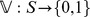. A solution to a constraint satisfaction problem is an 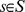 with 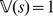. Note that here we are interested in obtaining the largest number of unique solutions to the constraint satisfaction problem. Formally, we want to maximize the cardinality of the set 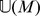, where 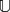 is a function that removes duplicates from the set of solutions 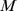 obtained using a search method, 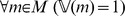. Although several deterministic methods exist to solve constraint satisfaction problems [14], they are usually designed with strong assumptions on the search space or constraints, e.g. only for linear constraints, or for solving specific problems, for example scheduling. As evolutionary algorithms are naturally apt to operate on non-linear objectives and constraints and maintain a population of solutions they are more suited for the specific problem tackled here, i.e. discovering not a single but multiple solutions to the constraint satisfaction problem.

Solving constraint satisfaction problems with evolutionary algorithms requires introducing a fitness function into the problem definition [13]. However, when using traditional evolutionary algorithms, the formulation of a suitable fitness function represents a major difficulty [15]–[17]. For example, when more than one objective must be simultaneously maximized or minimized or solutions must satisfy multiple constraints, the combination and weighting of multiple objectives and constraints into a single fitness function is challenging. Several multi-objective evolutionary algorithms, that do not require the aggregation of objectives into a single fitness function, have been proposed [18], [19], as well as techniques to handle problem constraints [20], [21]. However, little research has been devoted to methods capable of handling both objectives and constraints at the same time [22].

A difficulty of evolutionary algorithms that hinders the discovery of several unique solutions stems from the gradual loss of diversity caused by the repeated application of competition-based reproduction of the fittest individuals, which can lead to premature convergence of the evolving population to a sub-optimal solution [23], [24]. Furthermore, in multi-modal problems, where there are multiple global or local optima, possibly distributed over the solution space, evolutionary algorithms tend to converge to only one set of solutions (*i.e.* one global or local optimum) as population diversity decreases. Although several techniques have been proposed to delay or reduce premature convergence (see [25]–[27] for a review of existing methods), loss of population diversity is intrinsic to the majority of evolutionary algorithms and is influenced by the selection method employed. It has been recently mathematically proven that an optimal selection procedure for evolutionary algorithms consists of adaptively choosing a threshold on the fitness value so that all individuals with above-threshold values are selected for reproduction [28], [29]. However, the authors do not propose a method for automatic choice of suitable fitness thresholds.

Here we describe an alternative abstraction of artificial evolution, called Viability Evolution (ViE), which builds on thresholded fitness [28], [29] in the context of viability theory from dynamical systems theory [30], natural evolution [31]–[33] and behavior [34], to address the issue of fitness composition and premature convergence, potentially obtaining a larger number of unique solutions at completion of evolutionary process. An organism is called “viable” if it satisfies all the conditions defined on a set of critical physiological parameters, such as temperature range and energy levels, which define the viability boundaries of the organism [30], [31]. Similarly in ViE, individuals are deemed viable if they satisfy conditions defined by limiting boundaries on the problem, such as the range of allowed operating voltages of an electronic circuit or the feasible thickness values of a wing profile. As in the constraint adaptation approach [35], boundaries are initially set to enclose all individuals of the initial, randomly-generated population and are modified at discrete time steps. Individuals that fall outside the viability boundaries, either by means of mutations or by means of boundary modifications, are eliminated from the population. In this context, the viability boundaries can be seen as the threshold values of the fitness in the theoretical approach mentioned above [28], [29]. Boundaries are modified so that at least a user-defined fraction of the population is eliminated, and are maintained unchanged until the population grows back to at least the size before elimination. Thus, a variable population size is an inherent property of Viability Evolution. Since the population size can increase only by producing viable offspring, it can remain smaller than the initial size for many iterations after each viability elimination operation (for a more detailed discussion of viability theory in artificial evolution, see [36]).

Here we show that Viability Evolution maintains a higher level of diversity, leading to the discovery of a larger number of alternative solutions than found by traditional evolutionary algorithms without the definition of a compositional fitness function.

## Viability Evolution Algorithm

**Figure 1:**
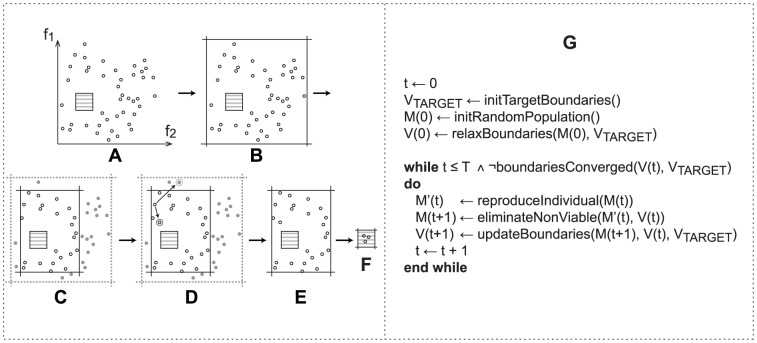
The Viability Evolution (ViE) algorithm. The population under evolution is shown in a two-dimensional objective space, defined by the 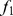 and 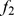 objective functions in this example. (A) Individuals of the initial population 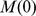 (black circles) are randomly generated. The region enclosed by the target viability boundaries (gray stripes) is extremely unlikely to contain any of the randomly generated individuals in the initial population. (B) The initial viability boundaries 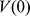 are set by the algorithm in terms of inequalities on the objectives to encompass all individuals in the initial population. (C) Viability boundaries are modified to approach the target boundaries 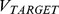; as a result, a fraction of the population becomes non-viable (gray shaded circles) and is marked for elimination. The way in which the boundaries are modified depends on the specific viability boundary update procedure implemented by the user. See Figure 2 for further details on the update mechanism used in this paper. (D) All viable individuals are allowed to reproduce by making one mutated copy at each iteration of the algorithm. Mutated copies that fall within the viability boundaries are allowed to stay along with the parent. Mutated copies that fall outside the viability boundaries are marked for elimination. (E) Non viable individuals are eliminated from the population. (F) The process described in (C–E) is repeated for many iterations until the viability boundaries reach the target values or the maximum number of evaluations 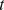 is exhausted. (G) The algorithmic description of Viability Evolution. Pseudo-code for the *relaxBoundaries* and *updateBoundaries* procedures is shown in Figure S8 and S9.

The Viability Evolution algorithm consists of defining viability boundaries, creating an initial population, and repetitively applying reproduction, elimination, and boundary updates until the boundaries meet the desired values (Figure 1). Viability boundaries are expressed as inequalities on the problem objectives and define the characteristics of the desired solutions.

**Figure 2:**
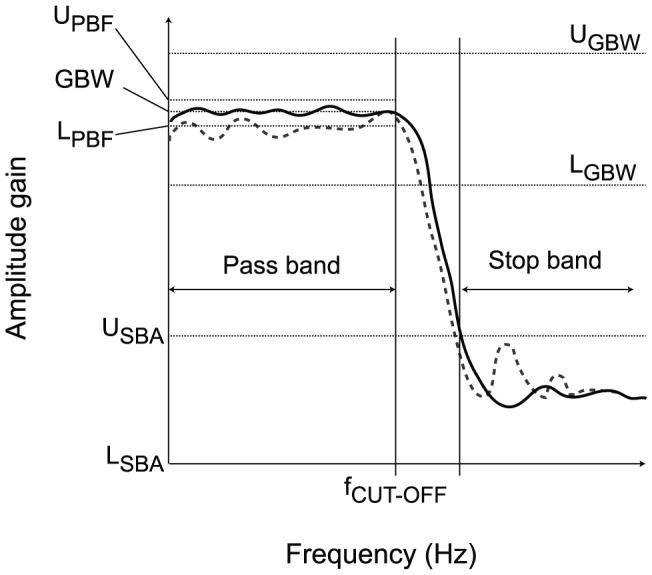
Example of viability boundaries definition for a filter design problem. A candidate filter design being optimized with Viability Evolution must satisfy certain requirements defined by the user as viability boundaries. Here, the filter gain-bandwidth product (GBW, computed at the cutoff frequency 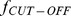) must satisfy the viability boundary 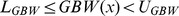. The stop-band attenuation (SBA) of the filter is also constrained by the viability boundary 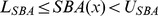. Finally, a filter must also satisfy a requirement on the pass-band flatness (PBF), i.e. the deviation of amplitude gain from the gain at cut-off frequency, such that 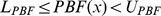. The response for two different filters is depicted in figure. The first filter (solid line) is viable as it satisfies all viability boundaries, while the second filter (dashed line response) is non-viable, as it violates the viability boundaries expressed on pass-band flatness.

To clarify the workings of Viability Evolution, let us consider the example of finding the electronic design of a low-pass filter that meets desired values for the gain-bandwidth product (GBW), the pass band flatness (PBF) and the stop band attenuation (SBA). These three parameters represent the viability conditions for the survival of the circuits. For each viability condition 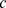 a lower bound 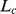 and an upper bound 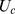 are defined (Fig. 2). A circuit 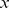 is deemed viable only if all its viability boundaries are satisfied, that is 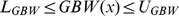, 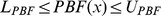, 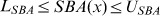. Because the initial population is randomly generated, it is extremely unlikely that any individual can satisfy all the viability conditions. Therefore, the lower and upper bounds of the viability conditions are initially set to encompass all individuals, and are gradually modified during evolution to approximate the desired values.

At each subsequent iteration of the algorithm, each individual can reproduce by adding a mutated copy to the population (the parent remains in the population). In order to give each unique individual in the population equal chance of being reproduced, we have to account for the possibility of clones, resulting for example from individuals that remain viable for a long time and produce lots of copies. To achieve this, the algorithm keeps track of the descendants of the initial population by assigning a different family identifier to every individual in the initial population (note that only mutations are used in reproduction). The reproduction probability of each individual takes into account the size of its family. This is done by first selecting a family of individuals inversely proportionally to its size from the current population and then randomly selecting an individual within that family (Figure S8). Once an individual has generated an offspring, its family identifier is assigned to the offspring and the family size is increased by one unit.

**Figure 3:**
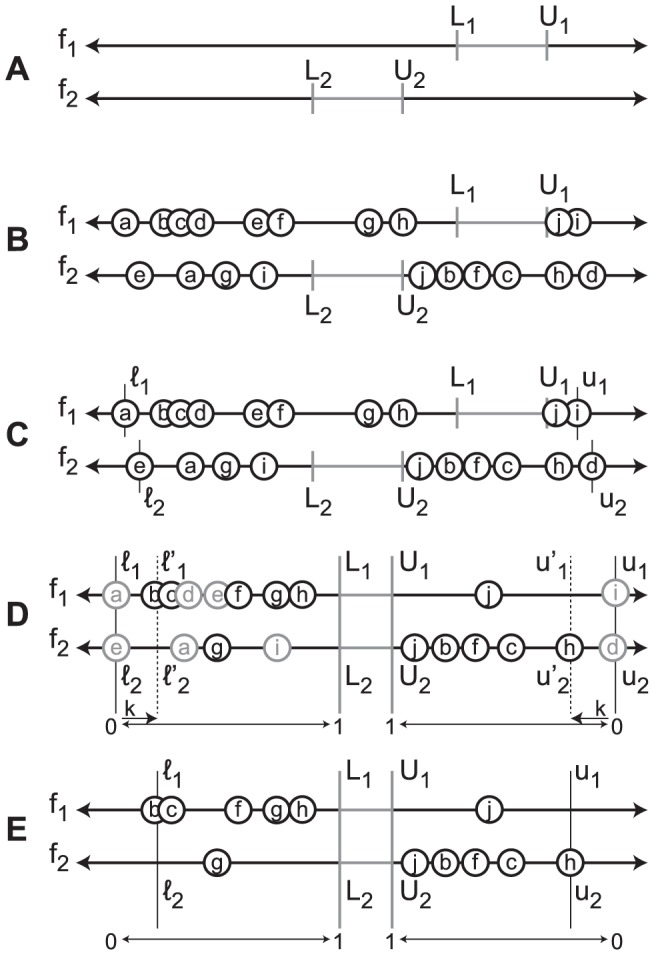
Boundary update mechanism in the Viability Evolution (ViE) algorithm. (A) Let us assume, without loss of generality, that the problem to be solved is defined by two objectives, 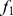 and 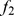. The target regions of the given problem are defined by the *target* viability boundaries - [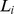, 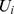] for each objective function 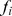 respectively. Thus, the goal is to find solutions which have values between 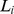 and 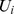 for each objective function 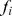 respectively. (B) Individuals of the initial population are randomly generated. Each individual is represented using a circle on the axis of each objective function. The position of a circle on the axis of an objective function 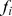 indicates the value of the corresponding individual for that particular objective. In this example, each individual is represented using 2 circles - one on each axis of the two objective functions. (C) The *initial* viability boundaries are set for each objective 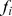 by identifying the extreme values [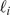, 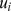] on either side of the corresponding target viability boundaries [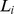, 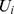]. The initial viability boundaries thus encompass all individuals in the initial population. (D) The viability boundaries are then tightened such that at least a minimum fraction 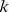 of individuals become non-viable. To illustrate this clearly, the intervals - [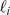, 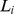], and [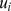, 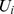] are both rescaled to [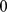, 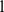] here. The new values for the viability boundaries [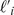, 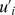] (shown as dotted lines) for each objective function 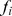 are computed such that at least a minimum fraction 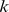 of individuals in the population become non-viable (shown as light gray circles). (E) Non viable individuals are eliminated from the population. The population continues to evolve with the new viability boundaries until the next boundary update. Pseudo-code for the boundary update procedure illustrated in figure is shown in Figure S9.

After the reproduction phase, individuals that fall outside the current viability boundaries are eliminated and the size of the families of these individuals is reduced accordingly. The two events that may lead to elimination of an individual are mutations and modifications of the viability boundaries. All the viability boundaries are modified simultaneously (Figure 3 and pseudo-code in Figure S9) so that at least a fraction of individuals (defined by the user) is discarded from the population. After each boundary update, boundaries are not modified until the population generates a number of viable individuals equal to at least the number of eliminated individuals. As soon as this condition is satisfied, the viability boundaries are updated again and this process is repeated until they reach the target values.

Once the boundaries are converged to the desired values, the algorithm returns the final population of solutions to the user. Note that all these solutions satisfy the user-defined criteria of success. Therefore, the user may choose any one of them or use additional criteria after inspection of the genotypes of the solutions.

## Results

We compared Viability Evolution on single-objective, multi-objective and constrained problems with two traditional, competition-based, evolutionary algorithms: a genetic algorithm with steady-state replacement [37], which will be denoted as SSGA in the rest of the paper, and the Elitist Non-dominated Sorting Genetic Algorithm (NSGA-II) [38].

Among the numerous evolutionary algorithms described in the literature, SSGA is the most similar to the ViE algorithm. Both SSGA and ViE follow the same cycle of parent selection, offspring generation, and selection of individuals for the next generation. Both use the same mutation operators to produce exactly one offspring per generation or iteration. SSGA and ViE differ in the mechanisms used to select the parent individuals for reproduction and the surviving individuals for the next generation. While SSGA uses the fitness-based rank of individuals for both operations, ViE allows all viable individuals to survive and reproduce. For multi-objective problems ViE was also compared against NSGA-II. NSGA-II is a widely used evolutionary algorithms for multi-objective optimization and uses sophisticated techniques to rank individuals and explicitly promote the maintenance of high diversity in the evolving population. All the three algorithms do not use crossover to simplify the analysis of the results.

**Figure 4:**
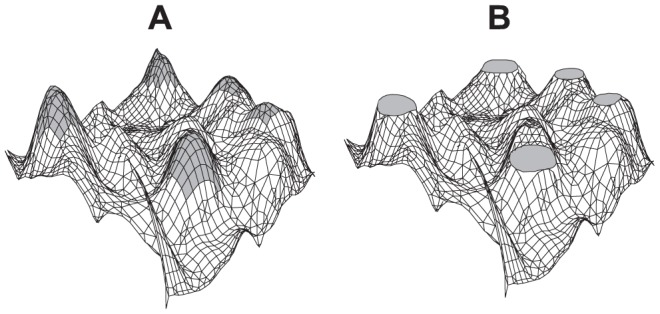
Thresholding of the fitness landscapes for single-objective problems. (A) A threshold on the fitness function of single-objective functions identifies one or more (possibly disjoint) target areas, depicted as gray regions. (B) In order to prevent competition-based algorithms from reducing diversity after reaching the target regions thus enabling a fair comparison to ViE, the fitness landscape is reshaped such that the same fitness value is assigned to any solution lying above threshold so that they all have the same probability of being selected for reproduction.

ViE and SSGA were compared on ten, single-objective optimization problems [39]–[42] (shown in Figure S1, their mathematical formulations can be found in Table S1). We decided to assess the performance of the algorithms on unconstrained single-objective problems to compare their ability to discover more diverse solutions in the simplest scenario as possible. In single-objective problems, the goal is to find the highest number of solutions with the best objective score or with a score that satisfies the user requirements. We compared genetic diversity and number of unique solutions discovered by the two algorithms. The number of unique solutions was measured as the number of unique individuals found within target areas defined on the benchmark functions. These target areas were specified by thresholding the objective functions (Figure 4A). All solutions with an objective value higher than the threshold were considered lying in a target area. The threshold value was defined so that the number of discoverable unique solutions was equal to the constant population size in SSGA and NSGA-II in order to ensure that ViE did not take advantage of its variable population size by simply increasing the number of viable solutions. To make the comparison fair, in SSGA we assigned the objective threshold value to all solutions within the target areas (Figure 4B) so that each had the same chance of being selected for reproduction, thus preventing the algorithm from further reducing diversity by selective reproduction of above-threshold solutions.

In some cases, the resulting target areas were disjoint (multiple global optima) and far away from each other (see Table S2 for the characterization of the different single-objective functions and the threshold values used), which represented some of the most interesting problems for the comparison of the two algorithms because their fitness landscapes contain very different target solutions (i.e., lying in the different target areas). For such problems, we compared the ability of the algorithms to thoroughly explore the solution space and find as many disjoint target areas as possible, while also considering the number of evaluations taken by each algorithm to find the target areas. The ten, single-objective problems included two functions with no local optima and single target area (Sphere and DoubleSum), three multi-modal functions with single target area (Rastrigin, Ackley, Langerman), and five multi-modal functions with disjoint target areas (FletcherPowell, Griewangk, Shubert, Vincent, Hump).

For multi-objective optimization, ViE, SSGA and NSGA-II were compared on three mathematical multi-objective problems (described in Tables S3 and S4), each composed of three objectives, obtained using a standard problem generator called DTLZ [43]. Moreover, the algorithms were compared on an electronic circuit design problem (Figure S5). The fitness capping method described above was also applied to NSGA-II.

Both SSGA and NSGA-II algorithms were terminated when the fitness values of all the individuals in the population reached the best achievable fitness. For ViE, which does not use a fitness function, this corresponds to terminating the algorithm when the viability boundaries reach the target boundary values. By limiting our experiments to two and three dimensional problems over finite solution spaces (bitstring encoding), we can enumerate the entire solution space and hence, precisely count the number of solutions in the target regions, which allows a precise comparison of the three evolutionary algorithms.

### Single-objective benchmarks

**Figure 5:**
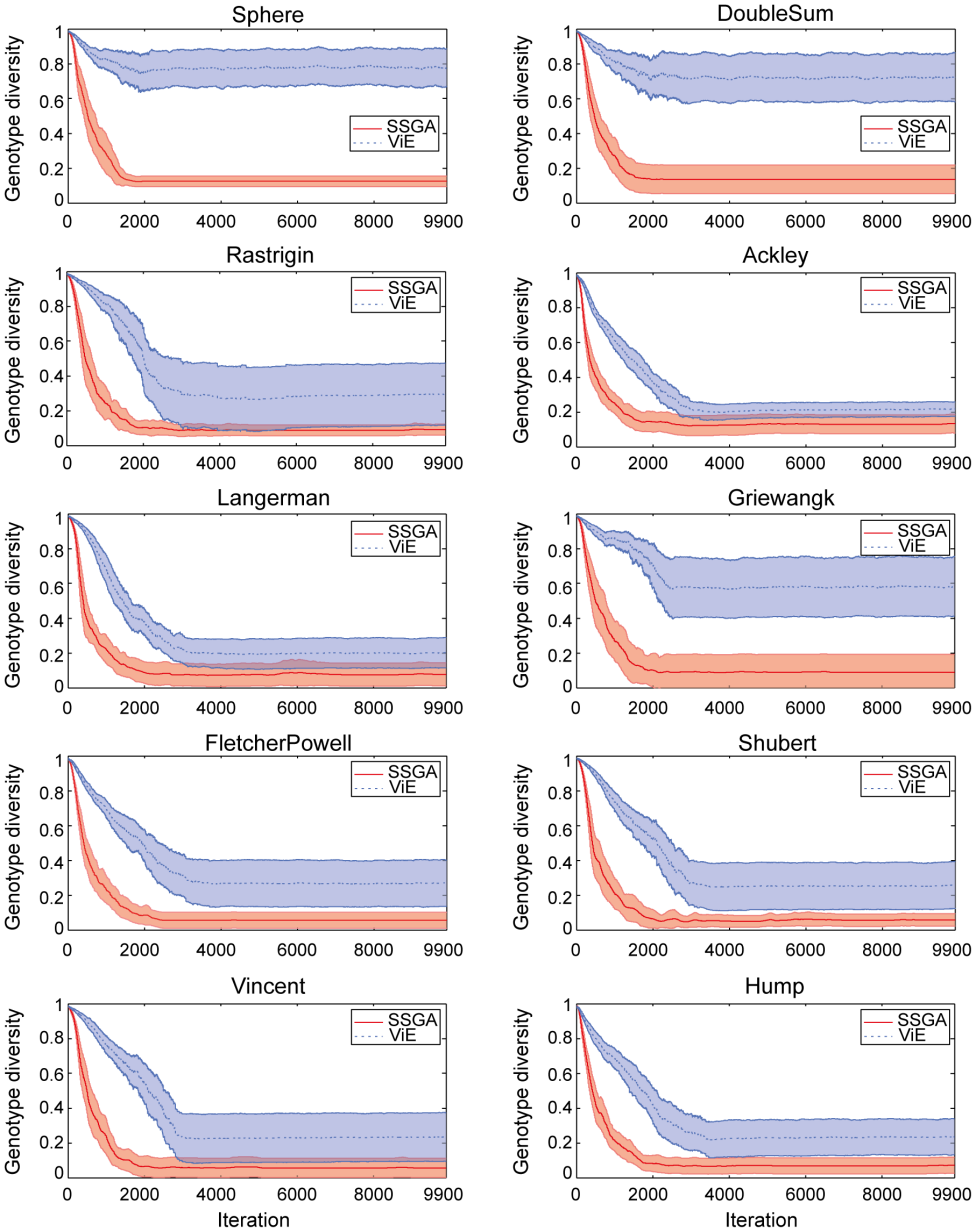
Genetic diversity maintained by SSGA and ViE. Average population genetic diversity (and confidence intervals) maintained during evolution for the 50 repetitions of each experiment.

**Figure 6:**
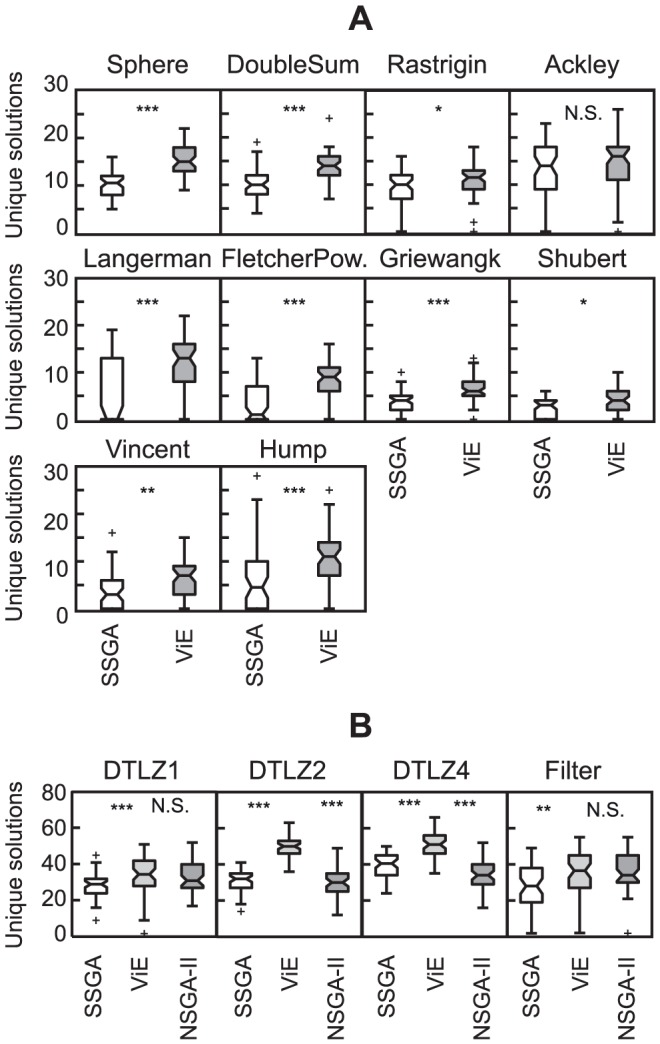
Number of unique target solutions discovered by SSGA and ViE. A) single-objective and B) multi-objective problems. Each boxplot shows results for 50 repetitions of the algorithms on each function (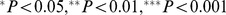, otherwise 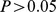, Wilcoxon rank-sum test; N.S. not significant).

**Figure 7:**
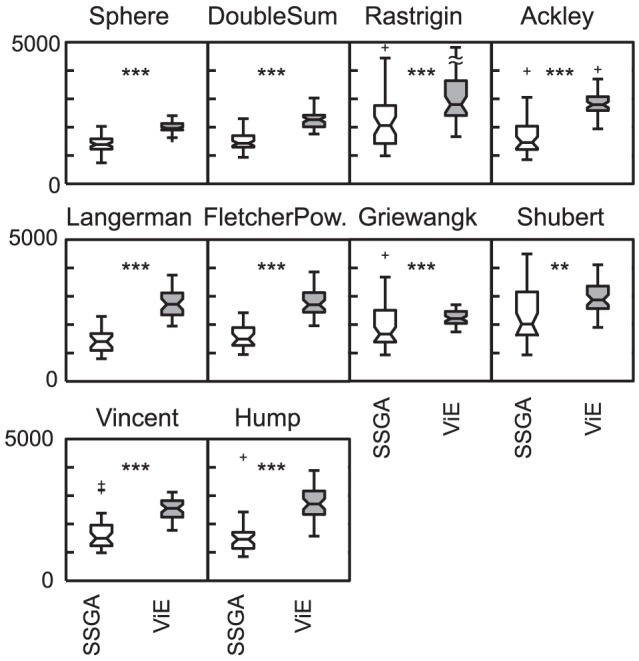
Number of iterations before completion of the evolutionary process for SSGA and ViE. Each box plot presents the results for 50 repetitions of the experiments on a different single-objective benchmark problem, as indicated by the titles above the boxes (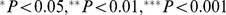, otherwise 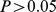, Wilcoxon rank-sum test; N.S. not significant). The maximum value of the ViE Rastrigin boxplot (not representable otherwise) is 5478.

ViE is able to maintain higher genetic diversity (based on Hamming distance between individuals as defined in [44]) than SSGA on all single-objective benchmark problems (Figure 5). Genetic diversity is significantly higher (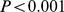, Wilcoxon rank sum test) over the entire evolutionary time, except for the initial iterations of the algorithms where diversity is comparable in both algorithms due to the random initialization of their populations. Higher genetic diversity in ViE results in a significantly higher number of unique target solutions in the population at the final iteration than in SSGA in all benchmark problems except Ackley where ViE display similar performance to SSGA (Wilcoxon rank sum test, Rastrigin and Shubert: 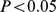; Vincent: 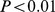; Ackley: 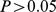, all remaining benchmark functions: 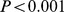), as shown in Figure 6A. This holds also for different values of mutation rates (see Figure S2 for genetic diversity, and Figure S3 for number of unique solutions). However, the better results achieved in terms of number of solutions discovered come at the cost of a longer evolutionary process for ViE in terms of number of evaluations before completion (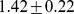 SD times longer than SSGA; see Figure 7 for non-aggregated results).

**Figure 8:**
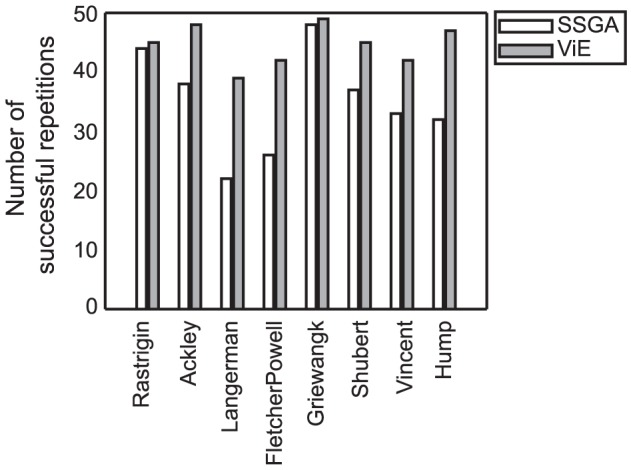
Number of successful repetitions for SSGA and ViE. Results for SSGA and ViE on single-objective, multi-modal problems out of a total of 50 repetitions.

The higher diversity maintained throughout evolution enables ViE to be more effective on multi-modal problems with respect to SSGA by escaping regions of the fitness landscape with local optima and eventually discovering regions with global optima. ViE always outperforms SSGA in terms of successful repetitions of the algorithm (Figure 8), defined as those repetitions where the algorithm discovers at least one target solution. SSGA prematurely converges and is not able to discover any target solution in many repetitions even though a low selection pressure was employed in these experiments (tournament selection with tournament size 2).

**Figure 9:**
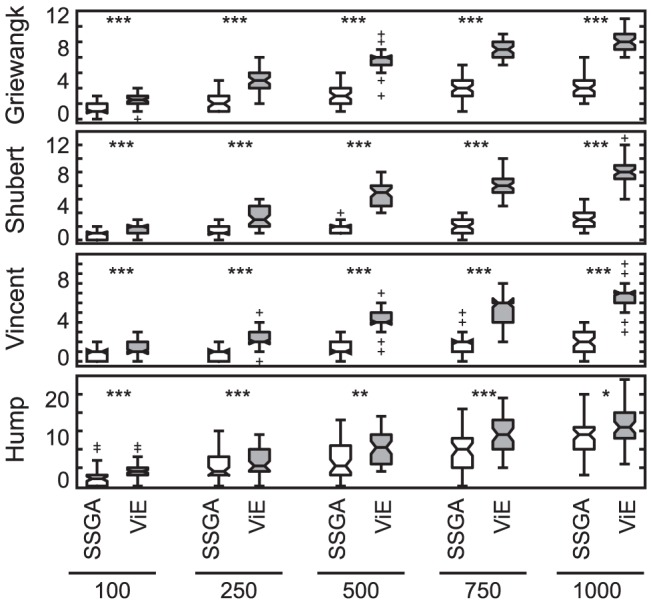
Number of disconnected target areas discovered by SSGA and Viability Evolution. Each box plot presents the results for different initial population sizes over 50 repetitions of the experiments (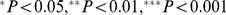, otherwise 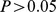, Wilcoxon rank-sum test; N.S. not significant). Viability Evolution can discover significantly more number of target areas for every initial population size (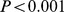, except Hump where 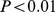 for population size 500 and 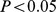 for population size 1000, Wilcoxon rank sum test) than SSGA.

The ability of ViE and SSGA to discover solutions in disconnected target areas within a single repetition of the algorithm was investigated on benchmark problems that feature a high number of disjoint target areas (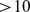, Griewangk, Shubert, Vincent, Hump). Furthermore, both algorithms were tested with different initial population sizes (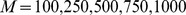 individuals) in order to assess if the algorithms could benefit from a larger, and potentially more diverse, initial population. ViE discovered more disconnected target areas than SSGA on all the benchmark problems and for all initial population sizes (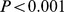, Wilcoxon rank sum test; Hump: 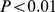; Figure 9).

**Figure 10:**
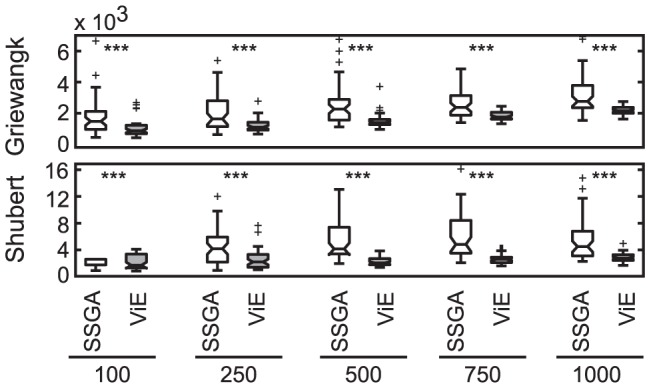
Efficiency of SSGA and Viability Evolution. Efficiency is measured as number of evaluations used per target area discovered over 50 repetitions of the experiment (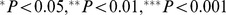, otherwise 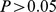, Wilcoxon rank-sum test; N.S. not significant). A repetition of the evolutionary experiment lasts a higher number of evaluations in Viability Evolution. However, Viability Evolution is able to discover more target areas per repetition than SSGA. Its efficiency is significantly better than SSGA (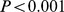, Wilcoxon rank sum test). To enhance readability of the box plots, we removed two outlier data points: Griewangk SSGA (500), Value 8676 and Griewangk SSGA (750), Value 12984. The computation of efficiency was performed only on Griewangk and Shubert, since the target areas in these benchmarks are regularly distributed in the search space and therefore have the same probability of being discovered.

Additionally, the efficiency of the two algorithms were compared as the average number of evaluations (individuals) necessary to find a single target area. ViE displayed significantly higher efficiency for all the tested initial population sizes (Wilcoxon rank-sum test, 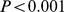; Figure 10).

### Multi-objective benchmarks and filter design problem

We compared ViE against the multi-objective optimization algorithm NSGA-II, which includes specific operators to maintain diversity in the evolving population. For sake of coherence with the results reported above, we also compared ViE and NSGA-II with SSGA endowed with a popular multi-objective technique, called weighted-sum approach [45], for combining multiple objective values into a single value. The three algorithms were assessed by counting the number of unique solutions that met the specified target performance for three mathematical functions and for an electronic circuit design. ViE performed better than SSGA on all the multi-objective problems (Figure 6B; Wilcoxon rank sum test, 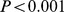 for DTLZ benchmarks, and 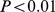 for the circuit evolution experiment). ViE performed better than NSGA-II on all mathematical problems (Wilcoxon rank sum test, 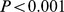, except for DTLZ1 where 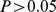) and performed as well as NSGA-II on the electronic circuit design (Wilcoxon rank sum test, 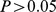).

## Discussion

In nature reproductive success depends on several factors that influence the probability of survival and reproduction of individuals. Two primary factors, as pointed out by Darwin([46], p. 116), are the competition among individuals for scarce resources (selection of the fittest) and the ability of individuals to withstand current environmental conditions (elimination of the non-viable). Traditional evolutionary algorithms are inspired by competition-based reproductive success by ranking individuals according to their fitness and selecting only the best for reproduction. The concept of elimination is seldom considered in Evolutionary Computation [47]–[49], and when it is, individuals are selected for elimination according to their fitness score, thus falling into the competitive scenario of reproductive success. Viability Evolution, instead, models reproductive success as the ability of individuals to withstand current environmental conditions and eliminates individuals that are not viable due to the effect of random mutations or changing environmental conditions (viability boundaries). The use of boundaries had been previously advocated to constrain evolutionary search in specific regions of the search space [35], but boundary update was based on competition among individuals rather than elimination. Viability boundaries can be seen as a set of binary fitness functions with adaptive thresholds [50], and in this perspective, here we provide a self adaptive procedure for threshold selection. It had also been suggested [51] that giving equal chance of reproduction to individuals satisfying a minimal fitness level could result in higher variability of the evolved solutions, but no practical algorithm was proposed. A threshold defining the survival of individuals was used in [52], but the threshold was always fixed to a constant value. This method was later extended [53], by progressively modifying the threshold. However, in both cases, the search was driven mainly by an objective promoting novelty of the solutions and the threshold was defined on a single objective. ViE does not use measures of novelty and drives the search by modifying viability boundaries on all problem objectives or constraints.

Viability Evolution can be used both for problem solving by defining target viability boundaries and for open-ended evolution by identifying viability boundaries that model the interactions between the evolving individuals and their environment (as in digital evolutionary ecosystems such as Tierra [54] and Avida [55], [56]). Novel environmental conditions could be easily introduced by adding or deleting viability boundaries at any time during the process of artificial evolution.

Even though the elimination step of the Viability Evolution algorithm resembles at first sight existing survivor selection methods employed in genetic algorithms (Culling Method [48], Truncation Selection [57], Extinctive Selection [2]) the resulting evolutionary dynamics of ViE are different (see Figure S4 for a practical example comparing SSGA with Truncation Selection to ViE) and are due to the interplay of eliminations, varying size populations, changing viability boundaries and the family mechanism. Using the insights obtained from the operational principles of Viability Evolution, one might construct a competition-based genetic algorithm with adaptive parameters (population size, fitness scaling, adaptive selection, etc.) to realize the properties of Viability Evolution and obtain similar performance. However, we believe that ViE's operational principle of “elimination of the non-viable” under changing viability conditions supplies the simplest model for an EA to achieve a performance as good as shown here.

To illustrate the advantages of the novel operational principle, we compared ViE to a canonical competition-based Evolutionary Algorithm, namely SSGA, without any state-of-the-art explicit diversity preservation techniques such as niching, maintenance of sub-populations, etc. One may argue that SSGA has not been designed for the specific problem domain considered here (i.e., maximize the number of unique solutions discovered at completion of the evolutionary process). Diversity preservation techniques might help SSGA achieve a higher number of unique final solutions. Thus, we compared Viability Evolution against SSGA endowed with a well-known diversity preservation technique, namely fitness sharing [58]. Viability Evolution can discover more unique solution than SSGA with fitness sharing in all benchmark problems (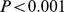; Shubert: 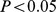, Wilcoxon rank sum test, Figure S6) except for Rastrigin, where results are not significantly different. Also, we tested viability Evolution against another technique which adds an explicit objective to foster diversity. The multi-objective method NSGA-II was modified to optimize two objectives: minimize the distance to the target areas, and maximize the diversity of the current population. This second objective was computed for each individual as the average Hamming distance between the individual and the other individuals in the population. Viability Evolution can discover a higher number of unique target solutions than NSGA-II with a diversity objective on all the benchmark problems (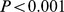, Wilcoxon rank sum test, Figure S7). It is possible that the application of fitness sharing in a steady state algorithm and the addition of an explicit objective for preserving diversity in NSGA-II may interfere with the search process. When using diversity preservation methods, one should consider that instrumenting an evolutionary method with such techniques usually requires the definition of additional parameters (for example a niching radius [59], or a niche capacity [60]), which are difficult to identify because the fitness landscape is unknown, or depends on measures of diversity in genotypic or phenotypic space [59]–[63], or requires keeping an archive of diverse solutions [61], [63]. Viability Evolution does not require the definition of additional niching parameters, diversity measures or the maintenance of an additional archive of solutions. Nonetheless, these explicit diversity preservation techniques are also applicable to ViE, and could possibly increase its performance too.

The family mechanism employed by ViE to prevent the dominance of clonal individuals may contribute to diversity preservation. To disambiguate the contribution given by the family mechanism we performed additional control experiments where we compared the number of unique target solutions discovered by SSGA, ViE, SSGA equipped with the family mechanism (SSGA-F) and Viability Evolution without the family mechanism (ViE-noF) on single-objective (Figure S10A) and multi-objective problems (Figure S10B). Both SSGA-F and ViE equipped with the family mechanism obtain equal or better performance than their versions without it (ViE-noF and SSGA). However, ViE can discover more unique target solutions than SSGA-F in four benchmark problems (Langerman and FletcherPowell: 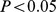; Hump and DTLZ2: 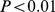, Wilcoxon rank sum test, Figure S10), and display performance similar to SSGA-F in the other benchmark problems. Also, ViE without family mechanism can discover more unique target solutions than SSGA on four benchmarks (FletcherPowell and DTLZ2: 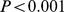; Griewangk: 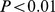; Rastrigin: 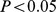, Wilcoxon rank sum test, Figure S10), and displays performance similar to SSGA in the other benchmark problems.

Although the dimensions of the problems in this study were kept small to make clear conclusions about the effectiveness of the EAs, the SSGA already fails to find target solutions in many runs (see Figure 8). Even though the scalability of the proposed approach to problems of higher dimensionality remains to be investigated, it must be considered that we presented here one of the possible procedures to update the boundaries (indeed a very simple one, to ease the comparison with respect to existing algorithms). The boundary update procedure presented here modifies all the boundaries together. This however is not a necessity as some boundaries may be harder to satisfy than others and may benefit from a differential update speed of each boundary. For example, each boundary update could be made proportional to the ratio of viable/unviable individuals for the corresponding objectives. In the future, more sophisticated procedures might be introduced, taking into account multiple factors to define which and by how much a viability boundary should be tightened (or relaxed), possibly enhancing the performance of ViE to address large-scale optimization problems.

Viability Evolution principles are applicable to several evolutionary algorithms. For example, the application of viability principles to CMA-ES [64], a state-of-the-art evolutionary method, beside providing an alternative method of handling multiple-objectives [65] or constraints [66], could also be an effective method for dynamically tuning the parent/offspring ratio (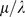 sampled individuals), and simplifying the offspring population resampling by giving equal weight to all viable individuals.

## Conclusion

Beside the better results in terms of number of unique solutions discovered by ViE on multi-modal and multi-objective problems (with the exception of the Ackley function and the electronic circuit design where ViE and NSGA-II reported the same performance), in Viability Evolution it is not necessary to aggregate multiple objectives or constraints into a single fitness function. Considering the well-known difficulty of designing fitness functions for multi-objective problems, this is a significant advantage even when ViE performs as well as other traditional evolutionary algorithms that require the formulation of an aggregated fitness function. When compared to multi-objective methods that do not aggregate fitness, ViE offers a different approach, which may even be applicable to those methods. Incidentally, the definition of viability boundaries in ViE is similar to the engineering practice of designing artefacts that meet desired operating ranges, such as temperature, voltage, frequency output, etc., which can be found in the specification list of any electronic or mechanical product on the market.

Although the main focus of this work is to show that artificial evolution can be performed with the sole use of viability based eliminations, ViE is compatible with the competition-based approaches and could be extended to encompass forms of competition-driven reproduction by introducing higher reproduction rates of viable individuals whose fitness could be computed while keeping unchanged all other aspects of the algorithm. A suitable combination of viability-based elimination and competition-based reproduction would allow a user to preferentially select for individuals with specific features within a diverse population of viable individuals and would provide a comprehensive evolutionary framework that models both competition and viability in natural evolution.

## Materials and Methods

Each evolutionary algorithm was assessed 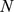 times (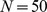) on each benchmark problem. For each repetition 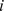 of an algorithm, the random number generator used by the probabilistic functions (i.e., generation of the initial population, reproduction, and mutation) was initialized using seeds 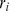, 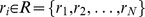, where 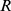 was a set of 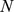 random numbers generated by software available at http://www.random.org/integer-sets/. The initial population size 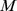 was set to 100 for the single-objective benchmarks and to 300 for the electronic circuit design problem and the 3-objective benchmark problems, unless otherwise stated in the Experimental Results section. For each repetition, we allowed each algorithm to evaluate at most 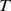 individuals (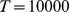), if the termination criteria were not reached earlier.

The genotype of the individuals was a binary string encoding 2 parameters for single-objective problems and for the electronic circuit problem, and 3 parameters for the multi-objective problems. Each parameter was encoded by 12 bits for single-objective problems, 10 bits for the electronic circuit problem, and 8 bits for multi-objective problems. Mutation consisted of flipping each bit of the genotype with probability 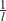 where 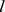 was the genotype length. In SSGA, selective reproduction was performed by means of tournament selection (size 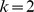, which corresponds to the lowest possible selection pressure). NSGA-II also employs tournament selection (size 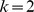) with the crowded comparison operator as proposed in [38]. Crossover was disabled in all the evolutionary algorithms. SSGA, and ViE generated 1 offspring per iteration while NSGA-II uses its default generational offspring generation and replacement policies. In the Viability Evolution algorithm, the fraction of killed individuals at every constraint update was set to 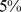 of the population size. The computer code, and all the software needed to reproduce the results presented in this paper can be found at http://lis.epfl.ch/VIE.

We used the NSGA-II multi-objective optimizer with constraints for the multi-objective experiments (available at http://www.iitk.ac.in/kangal/codes.shtml). The constraints were set to the target viability boundaries values. This ensures that the NSGA-II algorithm will attempt to maintain high diversity as well to reduce constraint violations, and correctly assign maximum preference to the solutions within the target area of the search space.

## Supporting Information

Figure S1 **Fitness landscapes for single-objective problems.** The single-objective functions include uni-modal, multi-modal and non-separable functions (Table S1). We defined fitness-capping thresholds on the landscapes to obtain a number of disconnected areas containing solutions at the same fitness level (Table S2). The Griewangk landscape, globally similar to Sphere, contains a large number of local minima that are indistinguishable in this figure.
(TIF)

Figure S2
**Genetic diversity of unique target solutions discovered by SSGA and ViE on single-objective problems, varying the mutation rate up to 10 times its original value.** Mutation, in the original configuration (1×), consisted of flipping each bit of the genotype with probability 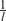 where 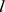 is the genome length. Each plot shows results for 25 repetitions of the experiments on each function. In general, genetic diversity increases with mutation rates. However, high genetic diversity obtained using high mutation rate does not always result into a higher number of discovered target solutions (Figure S3).(EPS)

Figure S3
**Number of unique target solutions discovered by SSGA and ViE on single-objective problems, varying the mutation rate up to 10 times its original value.** Each plot shows results for 25 repetitions of the experiments on each function. Mutation, in the original configuration (1×), consisted of flipping each bit of the genotype with probability 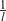 where 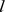 is the genotype length.(EPS)

Figure S4 **Average population genetic diversity (and confidence intervals) maintained by SSGA (with truncation selection) and Viability Evolution over 50 repetitions of the experiments.** Even though at first sight the update method used in ViE to tighten the viability boundaries may seem similar to SSGA with truncation selection (using an unusually high level of selection of 95% of the population), the evolutionary dynamics of these two algorithms are remarkably different.
(TIF)

Figure S5 **The filter design problem.** (A) A low-pass filter was evolved using the circuit topology derived from [67] (depicted in figure). This circuit topology allows the filter functionality to be modified using two bias current inputs (Bias-1 and Bias-2). The filter functionality is specified using constraints on three frequency response characteristics, namely gain-bandwidth product, pass band flatness and stop band attenuation. Hence, a solution to this problem is a pair of bias current values and the goal of an evolutionary algorithm is to find values for these two bias currents, assuming the fixed topology filter circuit, such that the specified low pass filter functionality is obtained. The three constraints on the frequency response characteristics of the filter are set such that there are approximately 300 (296, due to the quantization resolution introduced by the fixed bitstring encoding on possible values) bias current pair values that satisfy all three constraints. The performance of each candidate solution is obtained from simulations of the filter circuit using the SPICE circuit simulator. The SPICE models for the operational trans-conductance amplifiers (OTAs) used to build the filter circuit are available from http://www.ti.com/product/LM13700. (B) A typical frequency response of a low pass filter. The desired cutoff frequency f and output amplitude G are shown. The maximum deviation from G is defined by specifying a lower bound L and upper bound U. Finally, S represents the desired value for the maximum amplitude of any stop band ripple.
(EPS)

Figure S6
**Number of unique target solutions discovered by SSGA-FS and ViE on single-objective problems.** Each plot shows results for 50 repetitions of the experiments on each function (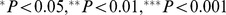, otherwise 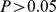, Wilcoxon rank-sum test; N.S. not significant). As SSGA was originally designed to discover optimal solutions and not to maximize the number of unique solutions discovered at the final generation, we equipped it with a traditional diversity preservation mechanism, fitness sharing [58], obtaining a modified version of SSGA named SSGA-FS. We set the niche-radius parameter 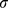 as suggested in [59]. Niche-radius values for each benchmark problems are reported in Table S5. The niche-radius is computed using 
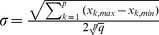
, where 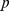 is the number of parameters, 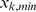 and 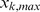 are the decision space boundaries of each parameter and 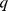 is the number of peaks (in our case disconnected target areas) in the fitness landscape. ViE can discover more unique solution than SSGA-FS in all benchmarks (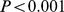; Shubert: 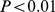, Wilcoxon rank sum test) except for Rastrigin, where results are not significantly different.(EPS)

Figure S7
**Number of unique target solutions discovered by NSGA-II with a diversity objective (NSGA-II-D) and ViE on single-objective problems.** Each plot shows results for 50 repetitions of the experiments on each function (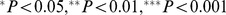, otherwise 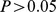, Wilcoxon rank-sum test; N.S. not significant). NSGA-II optimizes two objectives: 1) minimize the distance to the target area and 2) maximize the genetic diversity of each individual with respect to the current population, computed as average Hamming distance. ViE can discover a higher number of unique target solutions than NSGA-II-D on all the benchmark problems (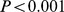, Wilcoxon rank sum test).(EPS)

Figure S8 **Pseudo-code for reproducing an individual in Viability Evolution.**
(EPS)

Figure S9 **Pseudo-code for the boundary update rule implemented of the Viability Evolution algorithm in this paper.**
(EPS)

Figure S10
**Number of unique target solutions discovered by SSGA, ViE, SSGA equipped with the family mechanism (SSGA-F) and Viability Evolution without the family mechanism (ViE-noF) on single- and multi-objective problems.** Each plot shows results for 50 repetitions of the experiments on each function (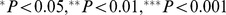, otherwise 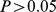, Wilcoxon rank-sum test; N.S. not significant). A) Single-objective problems results. ViE can discover more unique target solutions than SSGA-F in three benchmark problems (Langerman and FletcherPowell: 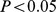; Hump: 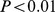, Wilcoxon rank sum test), displaying similar performance in the other benchmark problems. ViE-noF can discover more unique target solutions than SSGA on three benchmarks (FletcherPowell: 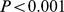; Griewangk: 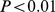; Rastrigin: 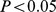, Wilcoxon rank sum test), displaying similar performance in the other benchmark problems. B) Multi-objective problems results. The contribution of the family mechanism always increases the performance of both SSGA and ViE respect to their versions without family mechanism (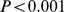, except when comparing ViE and ViE-noF in DTLZ1: 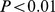, Wilcoxon rank sum test). Moreover, in the DTLZ2 problem, Viability Evolution can obtain better performance than SSGA both when comparing SSGA against ViE-noF (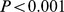, Wilcoxon rank sum test) and SSGA-F against ViE (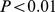, Wilcoxon rank sum test).(EPS)

Table S1
**Standard benchmark functions used to generate the single-objective fitness landscapes.** The 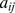, 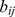 and 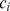 coefficients defined in the Fletcher-Powell and Langerman functions are the same used in [39]. The Hump function was randomly generated using the multimodal test generator presented in [68]. In the table we report the D-dimensional problem formulation (if available) or a 2-dimensional formulation. Furthermore, we denote if the functions employed are multi-modal (M) and/or separable (S), and their original reference (R).(PDF)

Table S2 **Characteristics of the fitness landscapes generated for the different single-objective experiments.** In this table, we report the benchmark function used to generate the landscape, the number of disconnected target areas (T) and the threshold applied on the original function to discriminate the target areas (A). Additionally, we classify these problems into three main categories: uni-modal with single target areas (a), multi-modal with single (b) or multiple (c) target areas, and indicate in the table which group each problem belongs to. The sum of the number of unique solutions over all the target areas of each problem is 100, except for Ackley (97).
(PDF)

Table S3 **The multi-objective DTLZ problem definitions.** The DTLZ problems, as originally introduced in [43], have been specifically designed for multi-objective EA and allow to control the difficulty of converging to the Pareto-optimal front. Specifically, these three problems pose different difficulties to the optimization algorithms. The DTLZ1 test problem requires the optimizer to find solutions on linearly distributed Pareto fronts, while the DTLZ2 and DTLZ4 test problems contain solutions distributed on spherical Pareto fronts. The DTLZ4 test problem has an additional problem difficulty as each front in the solution space contains an uneven distribution of solutions. Using ViE on multi-objective problems is simple because the experimenter does not have to combine the different objectives into a single fitness function, but can directly define the target set in terms of constraints on the different objectives (see Table S4 for the definition of the target viability sets).
(PDF)

Table S4 **The target viability boundaries for the multi-objective benchmark problems.** The target boundaries for the DTLZ and the filter design problems are described by constraints on the problem objectives. This table shows the target viability boundaries A and the number of target solutions M for each problem.
(PDF)

Table S5 **Niche-radius values for SSGA with fitness sharing in single-objective benchmarks.** The values are derived from the formula suggested in [59].
(PDF)

Text S1 **Additional experiments performed on Viability Evolution.**
(PDF)
