# Supplementary figures and images for: Artificial Evolution by Viability Rather than Competition
Source: PLoS One. 2014 Jan 29;9(1):e86831. doi: 10.1371/journal.pone.0086831 (PMC3906060; doi:10.1371/journal.pone.0086831)

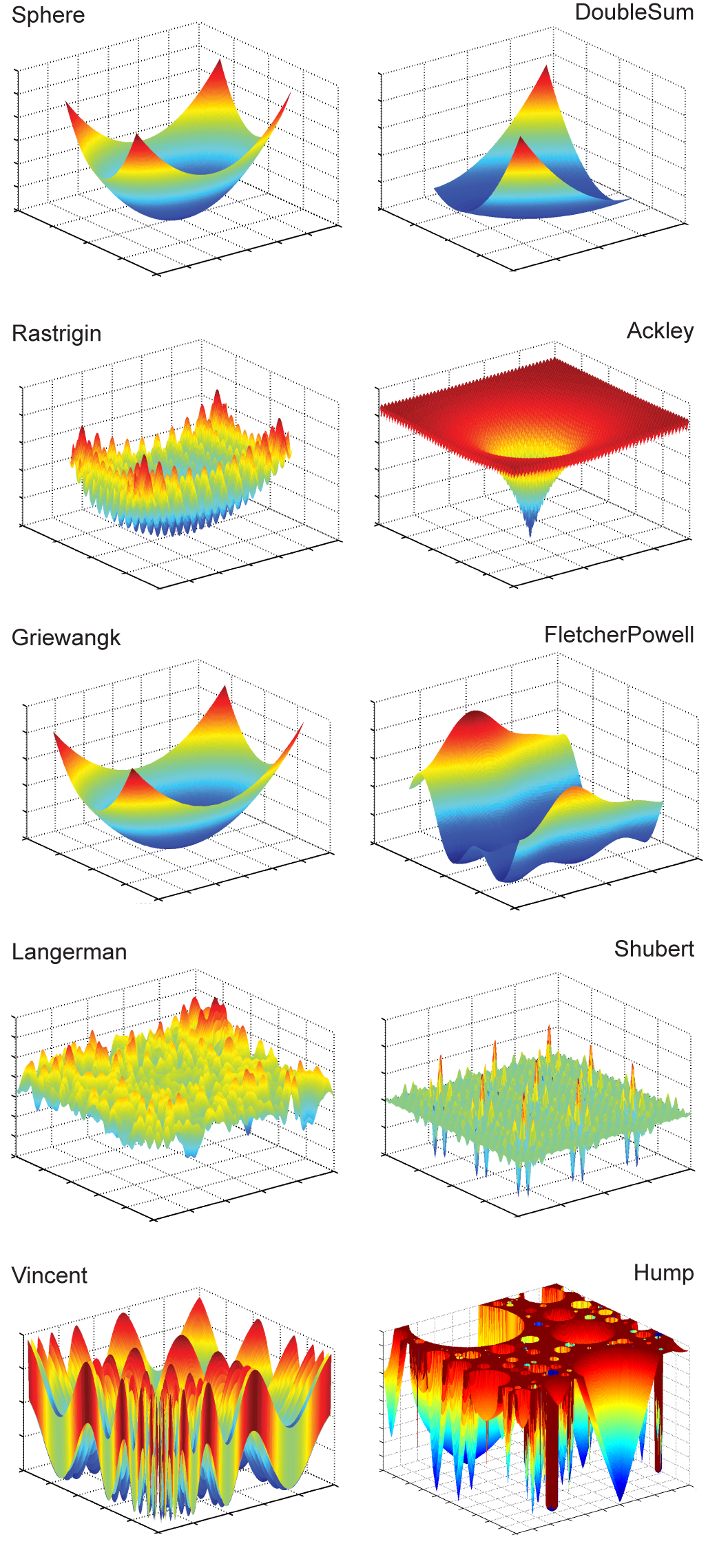

Supplement: Figure S1 — Fitness landscapes for single-objective problems. The single-objective functions include uni-modal, multi-modal and non-separable functions (Table S1). We defined fitness-capping thresholds on the landscapes to obtain a number of disconnected areas containing solutions at the same fitness level (Table S2). The Griewangk landscape, globally similar to Sphere, contains a large number of local minima that are indistinguishable in this figure. (TIF) [file pone.0086831.s001.tif]

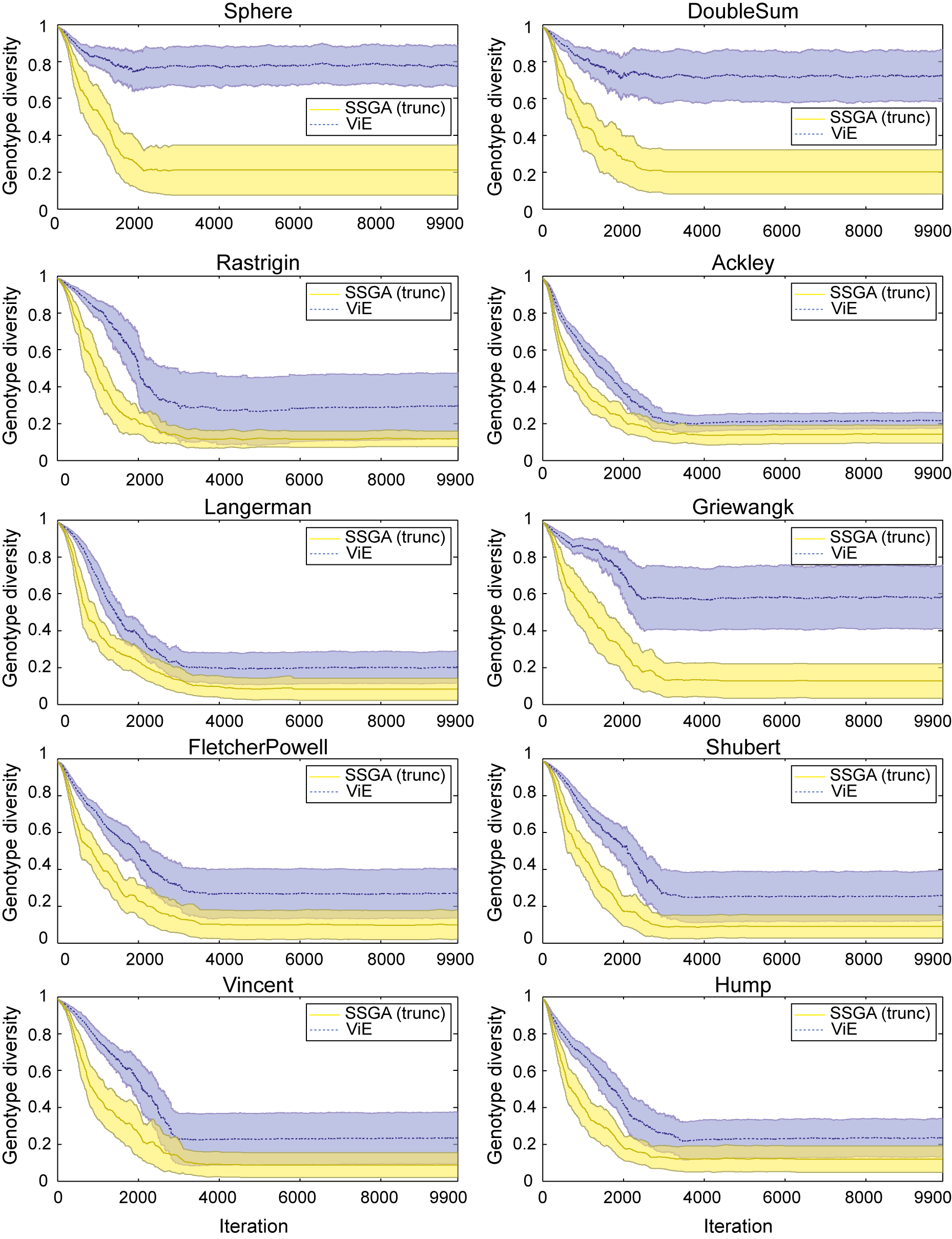

Supplement: Figure S4 — Average population genetic diversity (and confidence intervals) maintained by SSGA (with truncation selection) and Viability Evolution over 50 repetitions of the experiments. Even though at first sight the update method used in ViE to tighten the viability boundaries may seem similar to SSGA with truncation selection (using an unusually high level of selection of 95% of the population), the evolutionary dynamics of these two algorithms are remarkably different. (TIF) [file pone.0086831.s004.tif]
