# Supplementary material for: Artificial Evolution by Viability Rather than Competition
Source: PLoS One. 2014 Jan 29;9(1):e86831. doi: 10.1371/journal.pone.0086831 (PMC3906060; doi:10.1371/journal.pone.0086831)
Supplement: Table S1 — Standard benchmark functions used to generate the single-objective fitness landscapes. The , and coefficients defined in the Fletcher-Powell and Langerman functions are the same used in [39]. The Hump function was randomly generated using the multimodal test generator presented in [68]. In the table we report the D-dimensional problem formulation (if available) or a 2-dimensional formulation. Furthermore, we denote if the functions employed are multi-modal (M) and/or separable (S), and their original reference (R). (PDF) [file pone.0086831.s011.pdf]

Function	Formulation	Domain	M	S	R
Sphere	$f_1(\mathbf{x}) = \sum_{i=1}^{D} x_i^2$	$x_i \in [-5.12, 5.12]$		✓	[1]
Double Sum	$f_2(\mathbf{x}) = \sum_{i=1}^{D} \left(\sum_{j=1}^{i} x_j\right)^2$	$x_i \in [-65.536, 65.536]$			[1]
Rastrigin	$f_3(\mathbf{x}) = 10D + \sum_{i=1}^{D}(x_i^2 - 10 \cdot cos(2\pi x_i))$	$x_i \in [-5.12, 5.12]$	✓	✓	[1]
Ackley	$f_4(\mathbf{x}) = 20 + e - 20e^{\left(-0.2\sqrt{\frac{1}{D}\sum_{i=1}^{D} x_i^2}\right)} - e^{\left(\frac{1}{D}\sum_{i=1}^{D} cos(2\pi x_i)\right)}$	$x_i \in [-20, 30]$	✓		[1]
Griewangk	$f_5(\mathbf{x}) = 1 + \sum_{i=1}^{D} \frac{x_i^2}{400D} - \prod_{i=1}^{D} cos\left(\frac{x_i}{\sqrt{i}}\right)$	$x_i \in [-600, 600]$	✓		[1]
Fletcher-Powell	$f_6(\mathbf{x}) = \sum_{i=1}^{D}(A_i - B_i)^2$<br>$A_i = \sum_{j=1}^{D}(a_{ij} \cdot sin(\alpha_j) + b_{ij} \cdot cos(\alpha_j))$<br>$B_i = \sum_{j=1}^{D}(a_{ij} \cdot sin(x_j) + b_{ij} \cdot cos(x_j))$	$x_i \in [-\pi, \pi]$	✓		[1]
Langerman	$f_7(\mathbf{x}) = -\sum_{i=1}^{D} c_i e^{\left(-\frac{1}{\pi}\sum_{j=1}^{D}(x_j - a_{ij})^2\right)} \cdot cos\left(\pi \sum_{j=1}^{D}(x_j - a_{ij})^2\right)$	$x_i \in [0, 10]$	✓		[1]
Shubert	$f_8(\mathbf{x}) = \sum_{i=1}^{5} i \cdot cos\left((i+1)x_1 + i\right) \cdot \sum_{i=1}^{5} i \cdot cos\left((i+1)y_2 + i\right)$	$x_i \in [-10, 10]$	✓		[2]
Vincent	$f_9(\mathbf{x}) = -\frac{1}{D}\sum_{i=1}^{D} sin\left(10 \cdot log(x_i)\right)$	$x_i \in [0.25, 10]$	✓		[3]
Hump	$f_{10}(\mathbf{x}) = h_k \left[1 - \left(\frac{d(\mathbf{x},k)}{r_k}\right)^{\alpha_k}\right]$ if $d(\mathbf{x},k) \le r_k$ otherwise $f_{10}(\mathbf{x}) = 0$<br>$d(\mathbf{x},k)$ is the Euclidean distance to the k-th peak<br>where h_k, α_k and r_k are height, shape and radius of k-th peak	$x_i \in [0, 1]$	✓		[4]

References

[1] A. Eiben and T. Bäck, "Empirical investigation of multiparent recombination operators in evolution strategies," *Evolutionary Computation*, vol. 5, no. 3, pp. 347–365, 1997.

[2] J.-P. Li, M. E. Balazs, G. T. Parks, and P. J. Clarkson, "A species conserving genetic algorithm for multimodal function optimization." *Evolutionary computation*, vol. 10, no. 3, pp. 207–34, Jan. 2002. [Online]. Available: http://www.ncbi.nlm.nih.gov/pubmed/12227994

[3] O. M. Shir and B. Thomas, "Niche Radius Adaptation in the CMA-ES Niching Algorithm," in *Parallel Problem Solving from Nature - PPSN XI*, 2006, pp. 141–152.

[4] G. Singh and K. Deb, "Comparison of Multi-Modal Optimization Algorithms Based on Evolutionary Algorithms," in *Proceedings of the 8th annual conference on Genetic and evolutionary computation - GECCO '06*, 2006, pp. 1305–1312.
