# Supplementary material for: Artificial Evolution by Viability Rather than Competition
Source: PLoS One. 2014 Jan 29;9(1):e86831. doi: 10.1371/journal.pone.0086831 (PMC3906060; doi:10.1371/journal.pone.0086831)
Supplement: Table S2 — Characteristics of the fitness landscapes generated for the different single-objective experiments. In this table, we report the benchmark function used to generate the landscape, the number of disconnected target areas (T) and the threshold applied on the original function to discriminate the target areas (A). Additionally, we classify these problems into three main categories: uni-modal with single target areas (a), multi-modal with single (b) or multiple (c) target areas, and indicate in the table which group each problem belongs to. The sum of the number of unique solutions over all the target areas of each problem is 100, except for Ackley (97). (PDF) [file pone.0086831.s012.pdf]

**Benchmark**	**Function**	**Group**	**T**	**A**
Sphere	f_1	a	1	0.00020322
Double Sum	f_2	a	1	0.032016
Rastrigin	f_3	b	1	0.040295
Ackley	f_4	b	1	0.29747
Langerman	f_7	b	1	-3.0622
Fletcher-Powell	f_6	c	4	0.13806
Griewangk	f_5	c	13	0.14654
Shubert	f_8	c	18	-186.637
Vincent	f_9	c	36	-0.99998
Hump	f_{10}	c	36	-0.99951
