# Supplementary material for: Artificial Evolution by Viability Rather than Competition
Source: PLoS One. 2014 Jan 29;9(1):e86831. doi: 10.1371/journal.pone.0086831 (PMC3906060; doi:10.1371/journal.pone.0086831)
Supplement: Table S3 — The multi-objective DTLZ problem definitions. The DTLZ problems, as originally introduced in [43], have been specifically designed for multi-objective EA and allow to control the difficulty of converging to the Pareto-optimal front. Specifically, these three problems pose different difficulties to the optimization algorithms. The DTLZ1 test problem requires the optimizer to find solutions on linearly distributed Pareto fronts, while the DTLZ2 and DTLZ4 test problems contain solutions distributed on spherical Pareto fronts. The DTLZ4 test problem has an additional problem difficulty as each front in the solution space contains an uneven distribution of solutions. Using ViE on multi-objective problems is simple because the experimenter does not have to combine the different objectives into a single fitness function, but can directly define the target set in terms of constraints on the different objectives (see Table S4 for the definition of the target viability sets). (PDF) [file pone.0086831.s013.pdf]

Problem	Formulation
DTLZ1	$min\ f_1(x) = \frac{1}{2}x_1x_2...x_{n-1}(1+g(x_n))$
	$\ldots$
	$min\ f_{n-1}(x) = \frac{1}{2}x_1(1-x_2)(1+g(x_n))$
	$min\ f_n(x) = \frac{1}{2}(1-x_1)(1+g(x_n))$
	$g(x) = 1+(x-0.5)^2 - cos(20\pi(x-0.5))$
DTLZ2	$min\ f_1(x) = (1+g(x_n))cos(\frac{\pi}{2}x_1)...cos(\frac{\pi}{2}x_{n-1})$
	$min\ f_2(x) = (1+g(x_n))cos(\frac{\pi}{2}x_1)...sin(\frac{\pi}{2}x_{n-1})$
	$\ldots$
	$min\ f_n(x) = (1+g(x_n))sin(\frac{\pi}{2}x_1)$
	$g(x) = (x-0.5)^2$
DTLZ4	$min\ f_1(x) = (1+g(x_n))cos(\frac{\pi}{2}x_1^{\alpha})...cos(\frac{\pi}{2}x_{n-1}^{\alpha})$
	$min\ f_2(x) = (1+g(x_n))cos(\frac{\pi}{2}x_1^{\alpha})...sin(\frac{\pi}{2}x_{n-1}^{\alpha})$
	$\ldots$
	$min\ f_n(x) = (1+g(x_n))sin(\frac{\pi}{2}x_1^{\alpha})$
	$g(x) = (x-0.5)^2$
	$\alpha = 100$
