# Supplementary material for: Artificial Evolution by Viability Rather than Competition
Source: PLoS One. 2014 Jan 29;9(1):e86831. doi: 10.1371/journal.pone.0086831 (PMC3906060; doi:10.1371/journal.pone.0086831)
Supplement: Table S4 — The target viability boundaries for the multi-objective benchmark problems. The target boundaries for the DTLZ and the filter design problems are described by constraints on the problem objectives. This table shows the target viability boundaries A and the number of target solutions M for each problem. (PDF) [file pone.0086831.s014.pdf]

**Benchmark**	**A**	**M**
DTLZ1	$f_i(x) \in [0, 0.30937]$	296
DTLZ2	$f_i(x) \in [0, 0.58361]$	300
DTLZ4	$f_i(x) \in [0, 0.67827]$	300
Filter design	$c_1 \in [9 \cdot 10^6, 10 \cdot 10^6]$<br>$c_2 \in [0, 0.22]$<br>$c_3 \in [0, 0.2988]$	298
