# Supplementary material for: Artificial Evolution by Viability Rather than Competition
Source: PLoS One. 2014 Jan 29;9(1):e86831. doi: 10.1371/journal.pone.0086831 (PMC3906060; doi:10.1371/journal.pone.0086831)
Supplement: Table S5 — Niche-radius values for SSGA with fitness sharing in single-objective benchmarks. The values are derived from the formula suggested in [59]. (PDF) [file pone.0086831.s015.pdf]

**Benchmark**	**Niching radius**
Sphere	7.240773
Double Sum	92.681900
Rastrigin	7.240773
Ackley	35.355339
Langerman	7.071068
Fletcher-Powell	2.221440
Griewangk	235.339362
Shubert	3.333333
Vincent	1.149049
Hump	0.117851
