# Supplementary material for: Artificial Evolution by Viability Rather than Competition
Source: PLoS One. 2014 Jan 29;9(1):e86831. doi: 10.1371/journal.pone.0086831 (PMC3906060; doi:10.1371/journal.pone.0086831)
Supplement: Text S1 — Additional experiments performed on Viability Evolution. (PDF) [file pone.0086831.s016.pdf]

1 Supporting Information Text S1

To assess the performance of Viability Evolution we used standard single-objective benchmark functions introduced in [1, 2, 3] and multi-objective benchmarks from [4].

The single-objective functions included uni-modal, multi-modal and non-separable functions (Table S1). The different fitness landscapes for the single-objective problems are shown in Fig. S1. We defined fitness-capping thresholds on the landscapes to obtain a number of disconnected areas containing solutions at the same fitness level (Table S2).

The multi-objective problem definitions are listed in Table S3. We used a similar fitness-capping procedure by defining threshold values on the different objectives (Table S4).

1.1 Effects of mutation rates

In the main paper we analyzed the performance of the different algorithms on all the single-objective benchmark problem using a standard mutation rate of $\frac{1}{l}$, l being the genotype length in bits. In general, however, the optimal mutation rate for a given evolutionary algorithm depends on the selective pressure applied on the evolving population. In Viability Evolution, the selection pressure depends on the current problem difficulty, i.e. how easy it is to generate new viable-individuals. Therefore, there is an inherent difficulty in finding an optimal mutation rate for ViE as the selection pressure varies during evolution and the mutation rate should not be fixed as well. Moreover, in the main manuscript we set the parameter that regulates the amount of population eliminated due to the changing viability constraints to a small fraction of 5%, although it might be higher during the evolutionary process. Consequently, one might argue that the selection pressure in Viability Evolution is considerably lower than in SSGA and therefore the comparison under a non-optimal mutation rate of $\frac{1}{l}$ is unfair for SSGA.

We repeated the single-objective experiments and showed that even when varying the mutation rate, the results are consistent with what already discussed in the main paper. We changed the mutation rate up to 10 times the original value of $\frac{1}{l}$. Given the higher mutation rates, the time needed to converge increases as well. Hence, we set a maximum number of evaluations to 40000 for every tested mutation rate. Viability Evolution can consistently find a higher number of solutions than SSGA (Figure S3), with the exception of the Hump function), while maintaining a higher genetic diversity (Figure S2). As the mutation rate increases and reaches very high values, the algorithms do not converge anymore, as can be seen by the number of solutions (close to zero) and the very high genetic diversity.

1.2 Comparison against truncation selection

One might wonder if Viability Evolution is not a simple SSGA with truncation selection that employs an unusually high level of selection (truncation selection level at 95%), if one perceives the viability boundaries to be equivalent to the truncation threshold. However, such an analogy can be made only in single-objective problems. In a multi-objective scenario, to employ truncation selection one would need to compose the different objectives in a single fitness function, which is not needed in ViE. Second, truncation selection operates by selecting, for example 95% of the current population and generating offspring to fill the remaining 5% of the new population. This is a relative thresholding process that is applied to the current population and there is no check on the fitness values of the offspring that enter the new population. On the contrary, in Viability Evolution, the viability boundaries are specified on the absolute values of the objectives. The number of individuals eliminated from the current population will depend on the absolute values of their objectives rather than their relative values. Moreover, "non-viable" offspring cannot enter the new population. To further highlight the difference between Viability Evolution and SSGA with truncation selection, we performed an experiment comparing ViE and an SSGA with truncation selection at 95%. From Figure S4, it is clear that the evolutionary convergence characteristics of the two EAs are significantly different. In fact, the diversity levels maintained by Viability Evolution are higher than the ones obtained by SSGA with truncation selection.

References

[1] A. Eiben and T. Bäck, "Empirical investigation of multiparent recombination operators in evolution strategies," *Evolutionary Computation*, vol. 5, no. 3, pp. 347–365, 1997.

[2] J. Rönkkönen, X. Li, V. Kyrki, and J. Lampinen, "A Generator for Multimodal Test Functions with Multiple Global Optima," in *Simulated Evolution and Learning*, vol. 3, 2008, pp. 239–248.

[3] O. M. Shir and B. Thomas, "Niche Radius Adaptation in the CMA-ES Niching Algorithm," in *Parallel Problem Solving from Nature - PPSN XI*, 2006, pp. 141–152.

[4] K. Deb, L. Thiele, M. Laumanns, and E. Zitzler, "Scalable multi-objective optimization test problems," in *Evolutionary Computation, 2002. CEC'02. Proceedings of the 2002 Congress on*, vol. 1. IEEE, 2002, pp. 825–830.
